# DeepLeish: a deep learning based support system for the detection of Leishmaniasis parasite from Giemsa-stained microscope images

**DOI:** 10.1186/s12880-024-01333-1

**Published:** 2024-06-18

**Authors:** Eden Tekle, Kokeb Dese, Selfu Girma, Wondimagegn Adissu, Janarthanan Krishnamoorthy, Timothy Kwa

**Affiliations:** 1 School of Biomedical Engineering, Jimma University, Jimma, Ethiopia; 2 Pathology Unit, Armauer Hansen Research Institute, Addis Ababa, Ethiopia; 3 School of Medical Laboratory Sciences, Institute of Health, Jimma University, Jimma, Ethiopia; 4 Clinical Trial Unit, Jimma University, Jimma, Ethiopia; 5 Department of Chemical and Biomedical Engineering, West Virginia University, Morgantown, WV 26505 USA; 6 Medtronic MiniMed, 18000 Devonshire St. Northridge, Los Angeles, CA USA

**Keywords:** Leishmaniasis, Microscopic image, Deep learning, Object detection, Faster RCNN, YOLOV5, SSD

## Abstract

**Background:**

Leishmaniasis is a vector-born neglected parasitic disease belonging to the genus Leishmania. Out of the 30 Leishmania species, 21 species cause human infection that affect the skin and the internal organs. Around, 700,000 to 1,000,000 of the newly infected cases and 26,000 to 65,000 deaths are reported worldwide annually. The disease exhibits three clinical presentations, namely, the cutaneous, muco-cutaneous and visceral Leishmaniasis which affects the skin, mucosal membrane and the internal organs, respectively. The relapsing behavior of the disease limits its diagnosis and treatment efficiency. The common diagnostic approaches follow subjective, error-prone, repetitive processes. Despite, an ever pressing need for an accurate detection of Leishmaniasis, the research conducted so far is scarce. In this regard, the main aim of the current research is to develop an artificial intelligence based detection tool for the Leishmaniasis from the Geimsa-stained microscopic images using deep learning method.

**Methods:**

Stained microscopic images were acquired locally and labeled by experts. The images were augmented using different methods to prevent overfitting and improve the generalizability of the system. Fine-tuned Faster RCNN, SSD, and YOLOV5 models were used for object detection. Mean average precision (MAP), precision, and Recall were calculated to evaluate and compare the performance of the models.

**Results:**

The fine-tuned YOLOV5 outperformed the other models such as Faster RCNN and SSD, with the MAP scores, of 73%, 54% and 57%, respectively.

**Conclusion:**

The currently developed YOLOV5 model can be tested in the clinics to assist the laboratorists in diagnosing Leishmaniasis from the microscopic images. Particularly, in low-resourced healthcare facilities, with fewer qualified medical professionals or hematologists, our AI support system can assist in reducing the diagnosing time, workload, and misdiagnosis. Furthermore, the dataset collected by us will be shared with other researchers who seek to improve upon the detection system of the parasite. The current model detects the parasites even in the presence of the monocyte cells, but sometimes, the accuracy decreases due to the differences in the sizes of the parasite cells alongside the blood cells. The incorporation of cascaded networks in future and the quantification of the parasite load, shall overcome the limitations of the currently developed system.

**Supplementary Information:**

The online version contains supplementary material available at 10.1186/s12880-024-01333-1.

## Introduction

Image processing and artificial intelligence (AI) are pervasive nowadays in healthcare sectors by providing support to various diagnostic and therapeutic medical imaging modalities [1–3]. An accurate and timely diagnosis of diseases leads to a positive outcome for the patients treatment [4–6]. Thus, the current study aims to develop more sensetive diagnosing system for a Dermato-pathologic disease named Leishmaniasis by combining the best of image processing and AI domains.

Leishmaniasis is a vector-born neglected parasitic disease which is transmitted by the bite of infected female phlebotomine sand flies, and human infection is caused by 21 of the 30 known species [7, 8]. These are broadly divided into *Leishmaniasis donovani complex*, *Leishmaniasis Tropica*, *Leishmaniasis Mexicana complex*, *Leishmaniasis Aethopica*, *Leishmaniasis Majora*, and Subgenus *Vianna*. The disease is found in 88 countries, and approximately 350 million people live in Leishmania endemic areas (East and North Africa, India, Mexico, Central America, the Middle East etc.) [7, 9]. Besides this, it affects people living in areas associated with malnutrition, population displacement, poor housing, weak immune systems, and lack of financial resources [4]. Moreover, it is linked to environmental changes such as deforestation, the building of dams, irrigation schemes, and urbanization. Globally, an estimated 1.5 to 2 million new cases are reported every year, between 12 and 15 million people are infected, and 350 million people are at risk of infection. Besides, 70,000 deaths occur annually worldwide [9, 10]. The disease prevalence is not well studied/recorded at the country level. In Ethiopia, an estimated number of 4500 to 5000, new cases of Visceral Leishmaniasis (VL) are reported every year, and over 3.2 million people are at the risk of infection [11, 12].

**Fig. 1:**
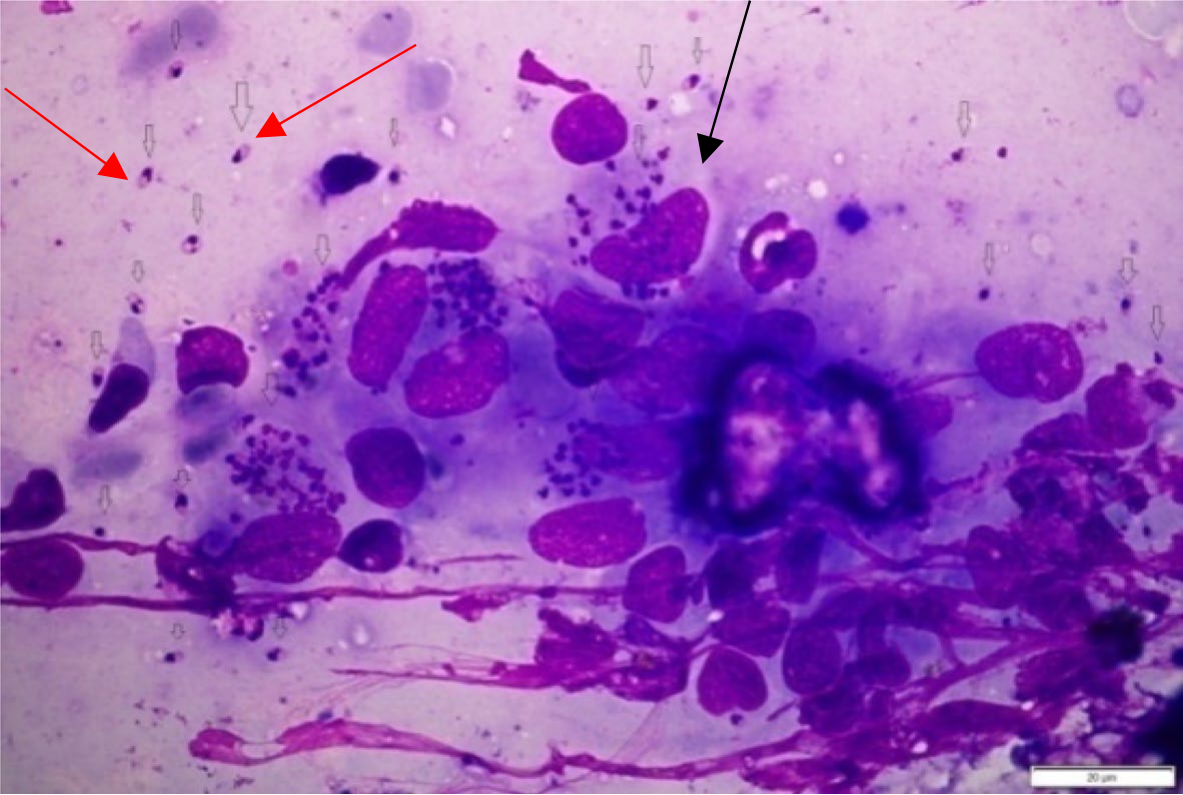
Microscopic image of Cutaneous Leishmaniasis (indicated by red arrows; the black arrow indicates monocyte)

Leishmaniasis manifests in three clinical forms: Visceral Leishmaniasis, Cutaneous Leishmaniasis (CL), and Muco-cutaneous Leishmaniasis (MCL). Visceral Leishmaniasis, also known as Kal-azar, proves fatal in over 95% of cases if untreated. Its symptoms include irregular fever episodes, weight loss, spleen and liver enlargement, and anemia. Globally, though an estimated number of 50,000 to 90,000 new cases of Visceral Leishmaniasis occurs annually, only around 25–45% of those cases are documented by the World Health Organization [8]. Visceral Leishmaniasis disease is mainly found around the northern regions of Ethiopia. Cutaneous Leishmaniasis stands out as the most prevalent variant of Leishmaniasis, characterized by the development of skin lesions, primarily ulcers, on exposed areas of the body. These lesions often lead to lifelong scars, contributing to significant disability or social stigma [13]. Mucocutaneous Leishmaniasis results in the partial or complete destruction of the mucous membranes in the nose, mouth, and throat [14]. Furthermore, it can also arise as a complication of Cutaneous Leishmaniasis (CL). Figure 1 illustrates the microscopic image of Cutaneous Leishmaniasis acquired at Armauer Hansen Research Institute (AHRI) for the purpose of this project.

Nowadays, incidence rate of the disease have started to increase at the country level [10, 15]. To diagnose Leishmaniasis, clinical observations, molecular methods, serological tests, and parasitological diagnosis are adopted in clinics [12, 16]. Yet, parasitological diagnosis remains the gold standard. It is carried out by taking a sample from the infected area and analyzing the presence of Leishman parasite (Leishman body). The presence of either the promastigote (extracellular, motile, early stage) or the amastigote (macrophage-intracellular, non-motile, late stage) of Leishman parasite through direct observation under a microscope. Detecting the parasite on a microscope requires skilled laboratory technicians, which is challenging in low-resourced developing countries. The procedure is generally tedious and time-consuming. The drug-resistance behavior of the disease is also another concern, making the effectiveness of treatments questionable [12, 17]. Besides, at the stage of amastigotes, the procedure is subject to false diagnosis because the parasite resembles other cells like neutrophils, platelets, bacteria, or some other artifacts found on the sample. To be consistent in the microscopic investigation, the expert laboratory technician has to go for 1000X views to claim a person is free from Leishmaniasis. Even if the molecular method provides accurate information, the device’s availability is limited to a few research institutions because of its expensiveness and the need for skilled laboratory technicians. Whereas, the serological test are preferred for rapid diagnosis because of its fastness and its ease of use. However, this test also provides unreliable (false negative/ positive) results.

A better diagnosing system facilitate both the treatment options as well as the disease-controlling options for the patients. So far only few studies have been carried out on Leishmaniasis that used both advanced image analysis and AI concepts. Yet, further investigations are required to improve the diagnostic procedures in reducing the unreliability in parasite detection. The primary objective of this study was to design and develop a deep learning-based decision support tool that can detect leishmaniasis parasites from Giemsa stained Microscopic Images and support the ongoing research in this area.

To develop the tool, we first acquired Giemsa-stained microscopic images of the parasite. We then trained different fine-tuned deep-learning object detection models based on these images to locate and identify the parasites. The developed tool was evaluated for its performance using various metrics, such as accuracy, sensitivity, and specificity.

## Related works

Recently, researchers have undertaken various investigations to address this issue. Some have attempted to develop image processing algorithms incorporating machine learning concepts for both the promastigotes and amastigotes stages of the parasite. However, these systems tend to be computationally expensive and primarily focus on classification tasks, lacking the ability to localize the target parasite. Conversely, a few researchers have endeavored to devise diagnosing methods based on deep learning. Nevertheless, as most of the datasets they utilized consist of fluorescent microscopic images, the practical application of such studies is confined to research centers. This underscores the necessity for the development of a more efficient Leishmaniasis diagnosing system. Researchers have gone through different investigations and experiments in microscopic image analysis for the last decades. Among the image processing approaches, P. A. Nogueira et al. [18] proposed a method using Otsu’s thresholding and Support Vector Machine (SVM) as a classifier and obtained 85.3% accuracy. The study used fluorescence-stained microscopy images from random drug trials with different experimental setups. Even if this method showed a good result, it failed to go for low parasite detection due to the segmentation step and did not quantify the parasite load. M. Farahi et al. [19] introduced another method called Modified Chan-vese (CV) Level Set method that automatically segments Leishmaniasis parasites from Geimsa-stained microscopic images. The study employed contrast stretching and mask production as a preprocessing step. Then, the CV model was used to extract the boundaries. To exclude the unwanted boundaries extracted on the sub-images, global and local methods were used and the study was able to achieve segmentation errors of 10.9% and 9.76%, respectively. In another study, J. C. Neves et al. [20] applied a blob detection method followed by contour detection and matching to classify the parasites in flurescence stained microscopic images. They were able to achieve an average F1 score of 84.48%. However, this method is complex and computationally expensive. Another approach proposed by F. Ouertani et al. considered the watershed segmentation technique combined with the region merging method [21]. Despite the initial segmentation and the merging step, some parasites remain improperly segmented. This is due to the overlapping cells and not considering the elliptical form of the parasite. This method can ensure the topological flexibility of the parasite, but it is computationally expensive because it requires knowledge about the shape and size of the parasite.

On the other hand, in the work by M. Górriz et al. [16], Leishmania parasites were segmented and classified into three groups using Fully Connected CNN and training using a U-net model that successfully segments Leishmaniasis parasites and classified them into promastigotes, amastigotes and adhered parasites. However, the study used a small number of fluorescent microscopic images (only 37 images), and there was a class imbalance within the datasets. M. Zare et al. introduced Viola Jones machine learning-based algorithm to classify Leishmaniasis parasites using 300 Geimsa-stained microscopic images. The research obtained 65% recall and 50% precision, giving a good indication and inciting that further investigations can be done [22].

Other methods used deep learning approaches on tropical infectious diseases like malaria, Trypanosomes. For example, O. Holmström et al. [23] evaluated the performance of deep CNNs on three different microscopy tasks: diagnosis of malaria in thick blood smears, tuberculosis in sputum samples, and intestinal parasite eggs in stool samples. In all cases, accuracy was high and substantially better than alternative approaches based on traditional medical imaging techniques. Hence, such systems can be used for automatic diagnosis of Leishmaniasis with larger datasets and better accuracy.

In the work by E. Yazdanparast et al. [24], a dedicated software called INsPECT was developed to automate infection level measurement based on fluorescent DNA staining. They also use morphological filtering as a pre-processing step followed by what they call a threshold for images with a Decreasing Probability Density Function.

As far as the literature review carried out in the current study is concerned, deep learning-based object detection algorithms for Leishmaniasis detection is not yet been developed. Believing that we are the first to apply deep learning models to detect leishmaniasis, we gave our system a pioneering and unique name: ‘***DeepLeish***’. However, for other parasitic and infectious diseases like malaria [25, 26] and tuberculosis, researchers introduced several object detection-based algorithms. For instance, J. Hung and A. Carpenter [27], in their study applied object detection algorithm on malaria microscopic images. The dataset contained 1300 images with 100,000 individual cells. The study used two stages: detection and classification. In the first stage, Faster RCNN was used to detect red blood cells only, and the output was fed into another network called ALEX Net with seven layers to classify the other cells, like parasites and leucocytes, into fine-grained categories.

## Materials and methods

### Study design and experiment

**Fig. 2:**
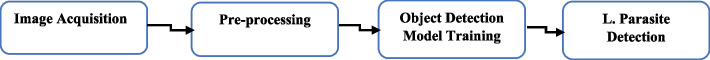
General block diagram of the developed system

The study was followed experimental research methods. Leishmania-infected Giemsa-stained microscopic slides were primarily obtained from the AHRI and Jimma Medical Center in Ethiopia. The control group (monocytes) was sourced from an online database. Microscopic images were acquired and labeled following secondary confirmation by clinical collaborators. The collected images underwent preprocessing before being inputted into deep learning-based object detectors to identify Leishman parasites. Figure 2 illustrates the general block diagram of the proposed method.

### Image acquisition

Figure 1 above shows acquired sample Geimsa-stained microscopic image of a Leishmaniasis case. The shape and color of Leishmaniasis parasites can be observed by the eye to identify the amastigote stage of the parasite. Amastigotes are round to oval shaped with 2–10 μm in diameter. The most prominent features are kenetoplast, larger nucleus, and cytoplasm. In the Geimsa-stained amastigotes stage of the Leishman parasite, the cytoplasm appears pale blue, and the kinetoplast appears pink and placed in front of the larger nucleus, which appears as a deep red color [28].

### Data preparation

**Fig. 3:**
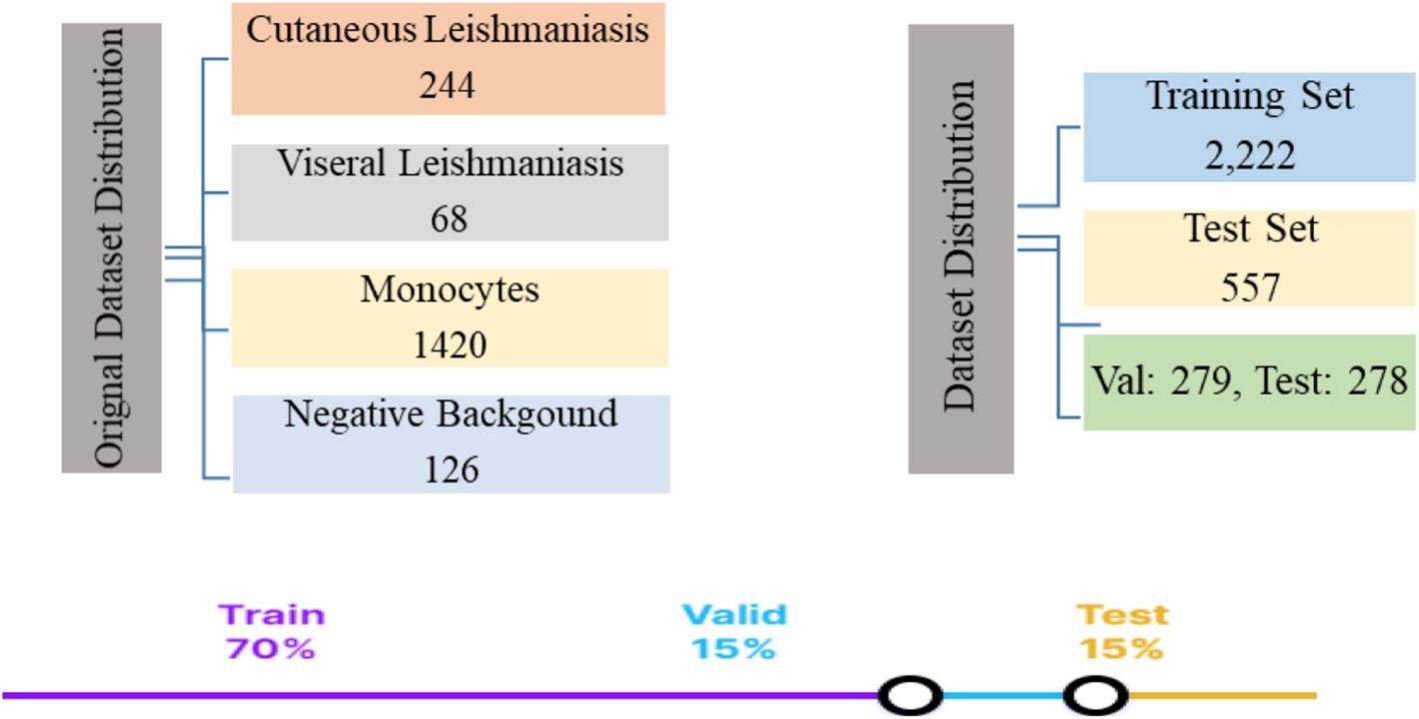
Dataset distribution and variability

Images were captured using a 12MP Olympus BX-63 digital microscope and a 5MP Olympus digital microscope from the two local sites. The online datasets were collected from medical images and the signal processing research center at Isfahan University of Medical Science, Iran (http://misp.mui.ac.ir/en/leishmania), and monocyte images were collected from an online Mendeley dataset [29]. In total, 1858 images were collected, out of which 244 are CL, 68 are Leishmaniasis parasites VL, 126 (negative background), and 1420 are monocytes. The data was split into 70% for training, 15% for validation, and 15% for testing. To increase the size of dataset, augmentation is performed (only on the training data set) and an additional 921 augmented images are obtained, making the total number of images 2779 out of which 2,222 images were annotated and used to perform training while the rest 557 images were used for validation and testing. The collected dataset considers the variability of the dataset. To elucidate, images containing parasite or monocytes only, images with concentrated parasites, and images containing both parasites and monocytes were considered. Both thin film and thick film stained microscopic images were considered as well. Figure 3; Table 1 present the data composition considered during training, validation, and testing. The data quality assurance followed by collecting the data is attached in the Supplementary material.

**Table 1:** Dataset distribution

Category	Number of Images
Original data	1858
Augmented data	921
Training Set	2222 (including augmented)
Validation Set	279
Testing Set	277

### Preprocessing

**Fig. 4:**
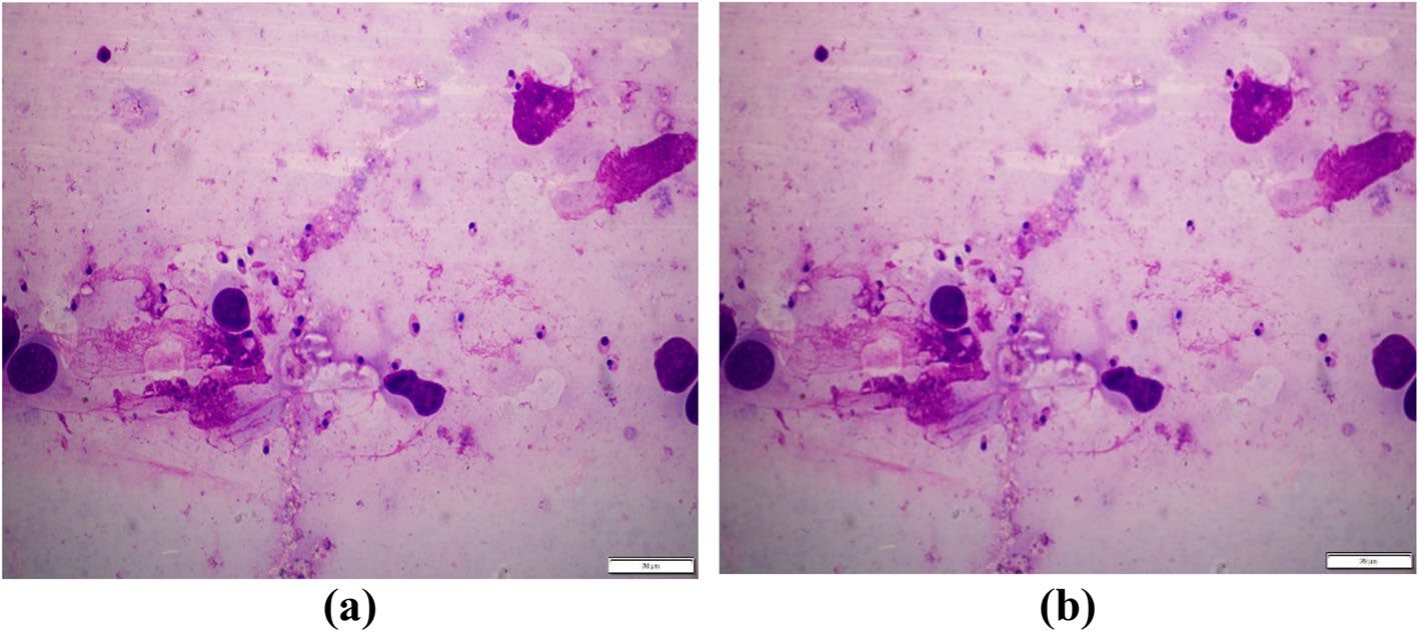
Image blurring using Gaussian filter. **a** Orignal image, **b** Gaussian blurring with a 3 × 3 kernel

Data augmentation, involving image rotation and Gaussian filtering, was utilized as a pre-processing step in the proposed method. This technique is employed to generate additional training data, particularly useful when dealing with small datasets, to facilitate the implementation of machine learning and deep learning algorithms [30]. In this study, rotation and Gaussian filtering methods were employed not only to address class imbalance but also to leverage several other advantages. Rotation, as a type of augmentation method, involves altering the orientation of the image across various angles. Essentially, it entails spinning the image around its axis to diversify the dataset and enhance model generalization [30, 31]. In this work, each image was rotated by 90^o^,180^o^, and 270^o^ (see Supplementary material). Another data augmentation technique utilized was Gaussian blurring. Gaussian noise is a common model used to simulate read-out noise in images, and Gaussian blurring, also known as smoothing, is employed to filter or reduce this noise [31]. Gaussian blurring is a method for generating multiple smoothed images by applying different kernel sizes without sacrificing essential information. Therefore, when developing deep learning-based object detection algorithms with limited datasets, Gaussian blurring serves as a crucial data augmentation technique. It helps assess the model’s ability to generalize and accurately detect the target object amidst the effects of blurring [32, 33]. Hence, without losing the essential features of the image, blurring (smoothing) with a kernel size of k = 3 × 3 was selected and applied to the original dataset (see Fig. 4).

### Model training & fine-tuning

Three deep-learning object detection models were chosen: Faster RCNN [27], YOLOV5 [34], and SSD ResNet [35]. The YoloV5 modelwas trained using 100 images containing leishman parasites with fine tunned hyper-parameters. A learning rate of 0.02 was used to train the model. A batch size of 128 was used, along with an anchor size of 3.44. After visualizing the output, the model was trained on all 2,222 images included in the training data set. The Faster RCNN model was trained with various Hyperparameters. An Epoch of 500 was chosen to train the model after performing trial and error experiments. A learning rate of 0.02 was used to train the model. In this work, a batch size of one (stochastic gradient descent) was used. Since the total dataset is not too large, such a small batch size was an option. In this work, a maximum of 100 detections were used, and other parameters remained the same as the default setting. Anchor boxes with aspect ratios of 1:1, 1:2, and 2:1, and scales of [0.25, 0.5, 1.0, 2.0] were selected in order to fit it to the anchor boxes with the target object. Similarly, for the third model, SSD ResNet50 FPN, training was done with different parameters where an Epoch of 500 and a batch size of 4 were finally assumed. Table 2 summarizes the Hyper-parameters used using the three deep learning schemes utilized in this work along with the computational resources required.

**Table 2:** Hyper-parameters and computational resources utilized

Model Type	Hyper-parameters			Computational Resource
	Learning Rate	Batch Size	Epoch	
YOLO_V5	0.02	128	1000	13.1 GB GPU
SSD	0.02	4	500	i7 CPU
Faster RCNN	0.02	1	500	i7 CPU

### Model evaluation metrics

To evaluate the detection performance of the different models, two major distinct evaluations are performed, which are nontrivial: classification loss and localization loss. Classification loss indicates how accurately the model classifies instances, while localization loss measures how precisely the model identifies the location of an instance. When dealing with datasets that have non-uniform class distributions, a simple accuracy evaluation metric may introduce biases. Therefore, analyzing the risk of misclassification is crucial. Thus, to measure the model at various confidence levels, it is necessary to associate confidence scores with the detected bounding boxes. Accordingly, the following evaluation metrics were computed and utilized: [26, 27].

**Precision**: is a measure of how predictions are accurate (given by Eq. 1). It is the ratio of correctly predicted positive observations to the total predicted positive observations (the sum of true positives (TP) and false positives (FP)).

1 $$Precision=\frac{TP}{TP+FP}$$

**Mean Average Precision (MAP)**: is the average value of average precision values (it is the area under the precision-recall curve) and is stated as Eq. 2 below.

2 $$mAP=\frac{1}{n}\sum\nolimits^{k=n}_{k=1}A{P}_{k}$$

Where n is the total number of classes, and AP_k_ is the average precision of class k.

**Recall**: is the ratio of correctly predicted positive observations to all observations in actual class (given by Eq. 3). It is defined in terms of TP and false negatives (FN).

3 $$Recall=\frac{TP}{TP+FN}$$

**Intersection over union (IOU)**: is an evaluation metric that is used to evaluate whether a predicted bounding box matches with the ground truth bounding boxes. Such evaluation metric is used to measure the quality of localization [36]. This is because, in the case of object detection, the (x, y) co-ordinate does not exactly match the ground truth due to the variation of our model (feature extractor, sliding window size, etc.). Thus, to verify the detected (x, y) coordinates indicate the exact position of the detected object, IOU is calculated. It implies the heavily matched bounding box will be rewarded. It is calculated as the ratio of the area of intersection to the area of the union of ground truth and predicted bounding boxes and given by Eq. 4 below.

4 $$IOU=\frac{Area\, of \,Intersection }{Area \,of \,Union }$$

## Results

### Detection evaluation results

The results obtained from the three models, i.e. YOLOV5, Faster RCNN, and SSD, are discussed in the next sections.

#### A. Detection performance of YOLO V5

**Fig. 5:**
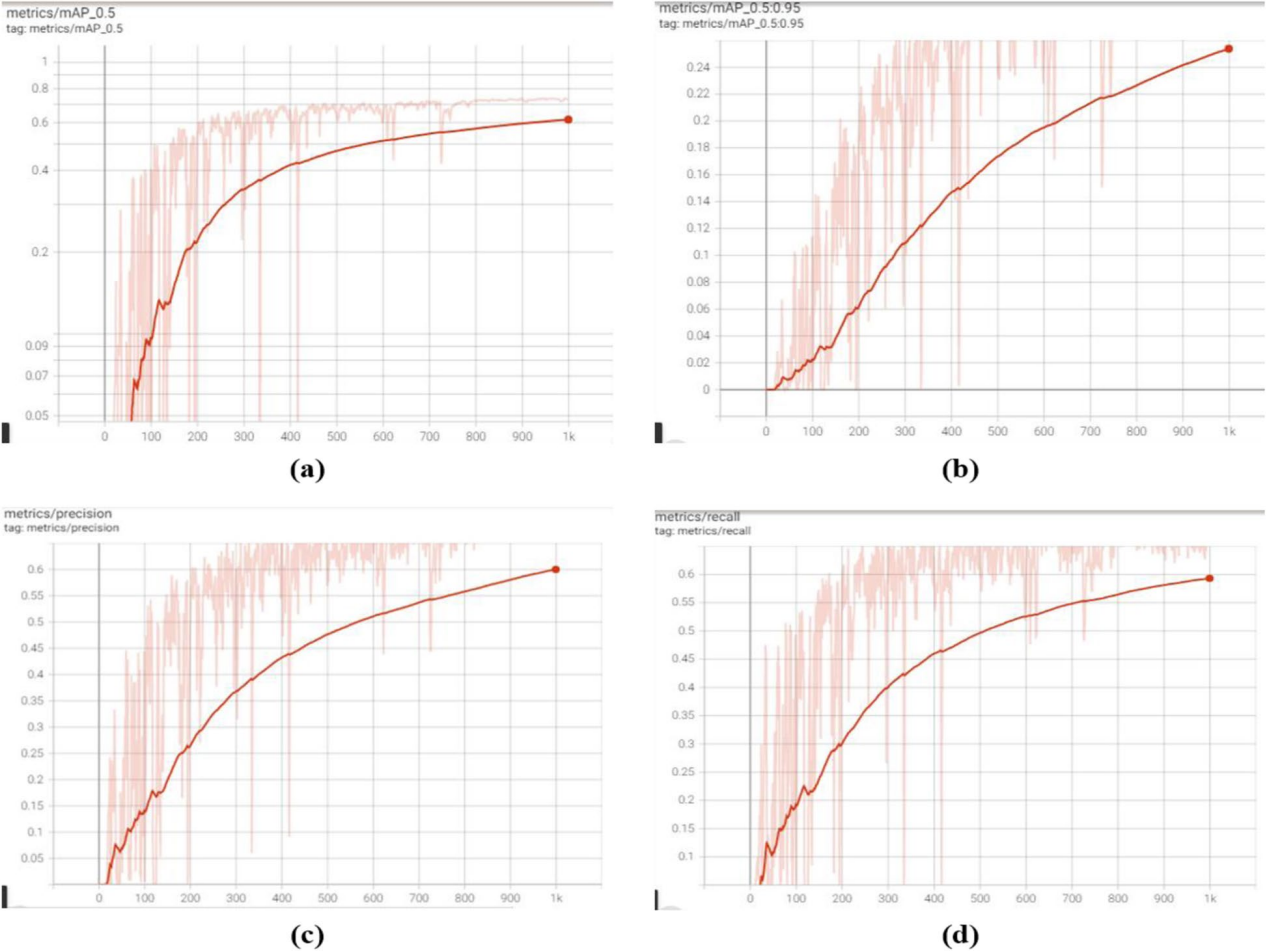
Evaluation results using YOLO V5: (**A**) MAP at IoU 0.5, (**B**) MAP at IoU 0.75:0.95, (**C**) Precision, (**D**) Recall

During the training of YOLOv5, approximately 13.1 GB of GPU memory was utilized to complete 1000 epochs on Colab, requiring several hours to run. The testing procedure averaged around 0.19 s per image, with the entire testing dataset taking approximately 54.15 s to process. The classification loss pertains to the box classifier, where the classifier identifies the targeted classes. The localization loss of the box classifier indicates the accurate localization of the targeted object. During the training phase, the box loss at 1000 epochs was 0.03998, and the objectness loss was 0.02118. Similarly, for validation, the box loss was 0.03377, and the objectness loss was 0.02185. As depicted in Fig. 5, the model was trained with different epochs and learning rates. The figure illustrates training with 1000 epochs and a learning rate of 0.01, as well as another training session with 500 epochs and a learning rate of 0.00014. The testing of the model was performed on Colab.

Table 3 presents the object detection performance of the model during training for selected learning rates, epochs, and batch sizes. The performance is evaluated in terms of MAP, Precision, and Recall. By increasing the learning rate from 0.00014 to 0.02 the error rate has decreased for YOLOv5.

**Table 3:** Performance of YOLO V5 for different epochs and learning rates during training

Experimental Setups	MAP	Precision	Recall
Learning rate : 0.00014 Epoch: 500 Batch size: 128	45.6%	55.5%	49.1%
Learning rate:0.01 Epoch: 1000 Batch size:128	63%	67%	67%
Learning rate: 0.02 Epoch: 1000 Batch size: 128	73%	68%	69%

The computed MAP values per a given IoU are presented in Table 4, which shows that a MAP of 65% is achieved. At the same time, the overall performance of the YOLO V5 model is summarized in Table 5, which achieved a better MAP, Precision, and Recall of 65%, 70%, and 69%, respectively.

**Table 4:** Evaluation results of YOLOV5 across different IoU values

Evaluation Based on IoU	IoU @0.5	IoU @ 0.75:0.95
MAP (Leishmaniasis parasites only)	73%	33%
MAP (with monocytes)	65%	35%

**Table 5:** Over all evaluation of YOLO V5

Overall Evaluation	MAP (@0.5)	Precision	Recall
Leishmaniasis Parasite Detection	73%	68%	69%
Leishmaniasis Parasite Detection including monocyte cells	65%	70%	69%

#### B. Detection performance of the SSD model

The SSD model was trained for about a month and was implemented in a local machine; compared to the YOLO V5 model, which was trained and tested using colab. The average time it took to detect parasites in test images was about 1.95 s. A sample result that demonstrate the performance of the SSD model is presented in Fig. 7, and the MAP values computed for different IoUs is presented in Table 6. The overall performance of the SSD model is summarised in Table 7.

**Table 6:** SSD evaluation results across different IoUs

Evaluation Based on IoU	IoU @0.5	IoU @ 0.5:0.95	IoU @ 0.75
**MAP**	56.3%	40%	43%

**Table 7:** SSD over all evaluation

Over all Evaluation	MAP (@0.5)	Precision	Recall
**Leishmaniasis Parasite Detection**	56.3%	40%	67.8%

**Fig. 6:**
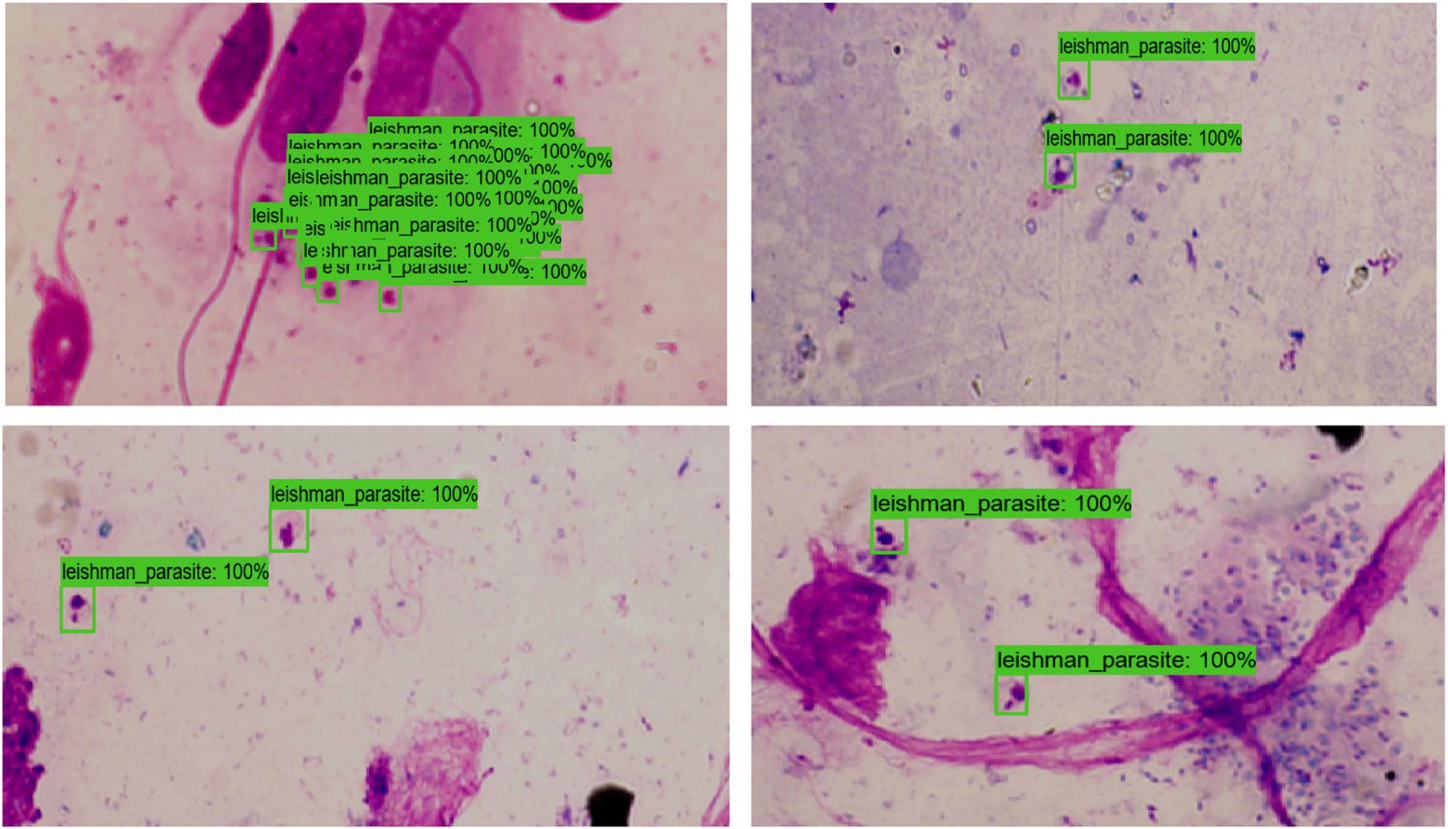
Shows test images detected by the SSD-trained model

Figure 6 below shows the detection performance of the SSD model for images containing Leishmaniasis parasites devoid of monocytes. The effect of the different levels of staining is evident from these captured images. The performance of the SSD model despite such variations in staining is shown as labelled parasites. Though not shown here, the performance of the model decreased relatively while identifying the parasite in the presence of the monocytes.

#### C. Detection performance of the faster RCNN model

Since Faster RCNN is a two-stage detection, first, the region proposal networks (RPNs) are generated. In the second stage, the overall classification and localization losses using the classifier (i.e. Faster RCNN) are computed. The classification loss is about the box classifier, in which the classifier identifies the targeted objects (classes). The localization loss of the box classifier indicates the proper localization of the targeted object. Table 8 below indicates the model evaluation results across different IOUs and its overall evaluation in Table 9.

**Table 8:** Faster RCNN evaluation results across different IoUs

Evaluation Based on IoU	IoU @0.5	IoU @ 0.5:0.95	IoU @ 0.75
**MAP**	54%	31%	35%

**Table 9:** Faster RCNN overall evaluation

Over all Evaluation	MAP	Precision	Recall
Leishmaniasis Parasite Detection	54%	31%	33.3%

### Comparison between candidate models

Table 10 presents the outcomes following the comparison performed between the three models (YOLO V5, SSD, and Faster RCNN) utilized in the current study in terms of their computational efficiencies during training and testing. In general, YOLO V5 was found to be computationally much cheaper than SSD and Faster RCNN, while the latter two models achieved comparable efficiencies. Note that YOLO V5 was implemented on colab while the other two models were run on a local machine, and the comparison in terms of computational efficiency may not necessarily be a fair one.

**Table 10:** Candidate models training and testing duration

Model	Training Duration	Testing Duration per Image
YOLO_V5	a day	0.19 s
SSD	a month	1.95 s
Faster RCNN	a month	1.8 s

**Fig. 7:**
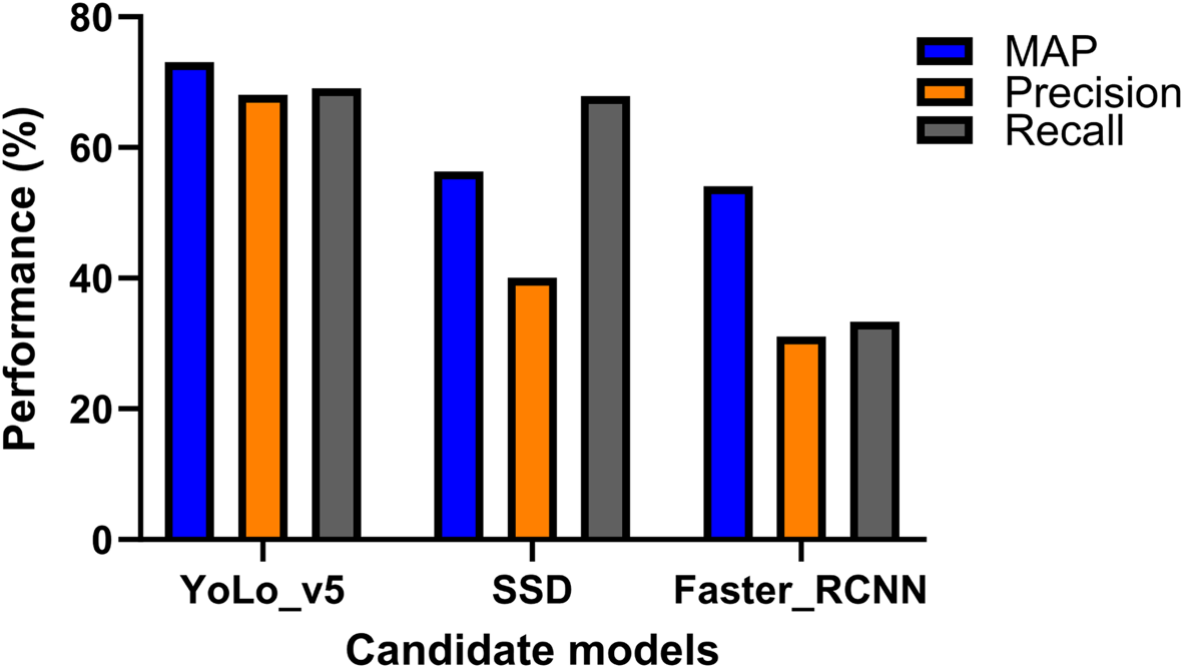
Performance comparison between the three models

Figure 7 above depicts bar graphs showing a comparison between the parasite detection performances in terms of the three matrices: MAP (at IoU of 50%), Precision, and Recall. Again, the highest MAP, Precision, and Recall values were recorded using YOLO V5. SSD performed better than Faster RCNN. In terms of Recall, the performance of YOLO V5 and SSD was almost comparable.

## Discussions

Table 11 summarizes the different studies carried out on Leishmaniasis parasite detection and compares their performance with our current study. In the list, three studies (including ours) used Geimsa stained microscopy images, while the rest used florescent dyes for detection purposes. Unlike our approach which relied on object detection, most of the other studies, relied on either segmentation or classification task. Though very sensitive and selective, the major limitation in adopting the fluorescence based approach in our study is the difficulty in sourcing the dyes as well as its availability in many of the clinics and research centers in sub Saharan region.

The previously reported study [22] that used machine learning for the detection of Leishmaniasis parasites from Geimsa-stained microscopic images, reported a lower precision and recall values of 50% and 60%, respectively, compared to our values of 68% and 69%, respectively. Further, that study was restricted to the design and development of an automated system for diagnosing Leishmaniasis disease using various transfer learning algorithms. This limitation is mainly attributed to the unavailability of larger dataset required for training the algorithm from scratch, as well as the constraints such as time and budget. Further, the researchers focused solely on the amastigote stage of the parasite and did not include the promastigote stage. Additionally, the identification of the causative agent (visceral or mucosal type) had not been addressed in this work.

The three pre-trained models: Faster RCNN, SSD and YOLO V5 considered in the current study used custom data for developing the computer-aided diagnosing system. The pre-trained Faster RCNN, SSD, YOLO V5 model based on the custom data achieved a MAP score of 54%, 56% and 73%, respectively, at 50% IoU. As mentioned earlier, the precision and recall of the current model is better than the previous reported study, at 50% IoU, while detecting the parasites in the presence of negative background and monocytes.

Previous studies have reported that generally, the YOLO V5 model outperforms other models when it comes to the identification of smaller objects [31, 32, 34]. Our experiment also confirmed that observation on YOLO V5 model that it could recognize the object of interest better than the other two candidate models. Further improvements in the efficiency of the YOLO V5 model came from the model’s capability to generate adaptable anchor boxes for the target object during training phase alongside the different augmentation techniques deployed for balancing the data in each class. Even though differentiating the Leishman body from the background image is challenging, the developed model was able to robustly and accurately detect the parasites.

**Table 11:** Comparison with recent findings

Author	Method	Types datasets	Results Obtained
**P. A. Nogueira et al.** [18]	Otsu thresholding; SVM classifier	Fluorescence microscopic image	85.3% classification accuracy
**M. Farahi et al.** [19]	Preprocessing (contrast stretching, masking); Segmentation (Modified Chan-vese (CV) Level Set Method)	Geimsa stained Microscopy images	Segmentation error of 10.9% using global CV and 9.76% using local CV
**J. C. Neves et al.** [20]	Blob detection based classification	Fluorescence microscopic image	Average F1 score of 84.48%
**F. Ouertani et al.** [21]	Watershed segmentation technique combined with region merging	Fluorescence microscopic image	NA
**M. Górriz et al.** [16]	U-net model classification	Florescence-stained microscopic image	NA
**E. Yazdanparast et al.** [24]	INsPECT (infection level measurement); Preprocessing (morphological filtering)	Fluorescent DNA staining	NA
***M. Zare et al***. [22]	Machine learning based Leishman parasite detection	Geimsa-stained microscope images	Precision 50% recall 60%
**Proposed Model**	**Object detection with YOLO_V5**	**Geimsa-stained microscope images**	**MAP 73%, Precision 68%, Recall 69%**

As depicted in Table 11 above, only our model developed using deep learning approach for Giemsa-stained microscopic images, has achieved a higher accuracy with a mean average precision (MAP) of 73%, precision of 68%, and Recall of 69%.

## Conclusion

The microscopic examination remains the gold standard method for diagnosing Leishmaniasis. However, this method imposes a significant burden on laboratory technologists, often resulting in misdiagnoses and time-consuming examinations. The current study address this issue by developing a computer-aided system that reduces the workload of pathologists and laboratory technologists by automatically detecting the Leishmaniasis parasites. Giemsa-stained microscopic images were collected, augmented and resized during pre-processing step. The pre-processed images were used for training three deep learning networks—YOLO V5, SSD, and Faster RCNN. The results during test phase indicated that YOLO V5 exhibited the highest detection capability, achieving a mean average precision (MAP) of 73%, precision and recall rates of 68% and 69%, respectively. The YOLO V5 model outperformed the other two candidate models and was proficient in identifying both monocytes and the Leishman body. Our system can expedite the diagnosis process, decrease misdiagnoses and significantly reduces the burden on laboratory experts. In future, robust preprocessing algorithms such as CutMix, MixUp, and AutoAugment; and other deep learning algorithms such as variants of EfficientNet, and vision transformer (SegFormer), shall be explored on our dataset to improve the efficiency of parasites detection. Additionally, larger dataset will be collected to train models that extends our current model through identification of the types of Lishmanianis such as Viceral or Cutaneous or Mucosal. Other aspects such as the severity of the infection or the parasite load will also be considered. Identification of secondary infections besides Leishmaniasis could also make our system more versatile and multifunctional. Such systems have interesting prospectus in telemedicine applications, particularly in low-resourced hospitals with inadequate number of experts available for diagnosis.

### Supplementary Information

Supplementary Material 1.

## Data Availability

The locally collected data and source code is available at 10.17605/OSF.IO/X7QHS.

## Abbreviations

AHRI: Armauer Hansen Research Institute
AQ-DAT: Aqueous antigen direct agglutination test
CL: Cutaneous Leishmaniasis
CNN: Convolutional Neural Network
Derm.patho.: Dermato-pathologist
ELISA: Enzyme-linked immune sorbent assay
FD-DAT: Freeze-dried direct agglutination test
FAST: Fast Agglutination Screening Test
IFAT: Immune-fluorescent antibody test
IHA: Indirect hem-agglutination test
Lab.Tek: Laboratory Technologist
MAP: Mean Average Precision
MCL: Muco-Cutaneous Leishmaniasis
MP: Mega Pixel
PKDL: Post Kal-azar Dermal Leishmaniasis
Rk39-ICT: rK39 immunochromatographic test
ROI: Region of interest
SVM: Support Vector Machine
VL: Visceral Leishmaniasis
