# Supplementary material for: DeepLeish: a deep learning based support system for the detection of Leishmaniasis parasite from Giemsa-stained microscope images
Source: BMC Med Imaging. 2024 Jun 18;24:152. doi: 10.1186/s12880-024-01333-1 (PMC11186139; doi:10.1186/s12880-024-01333-1)
Supplement: Supplementary file 1 — Supplementary Material 1. [file 12880_2024_1333_MOESM1_ESM.docx]

**Supplementary Material**

1. **Data Quality Assurance**

To verify the quality and reliability of the data,

1. Clinical collaborators and data collectors took an orientation about the overview of the research along with the specific task expected from them.
2. Expert dermatopathologists, pathologists, and laboratory technicians label the data containing Leishmaniasis images collected from an online database, ALERT/AHRI Center, Jimma Medical Center.
3. The collected data was handled, stored, and processed carefully in a confidential way.

In addition, a data health check was performed to avoid class imbalance and to understand the target objects' distribution. This is useful information to decide the preprocessing steps without losing important information. The average image size ranges from 0.17mp to 1.92mp, with a median image size of 416 X 416 as illustrated in Figure 4. To avoid class imbalance, a total of 6471 objects were incorporated. This includes adhered parasites, concentrated parasites, and parasites engulfed by macrophages.

Figure 1: Dataset health check.

1. **Augmentation Method**

**Rotation**

In this work, each image rotated by 90,180, and 270 degrees (see also Figure 2 and Figure 3 below).

Original image Rotation by 90^0^ Rotation by 180^0^ Rotation by 270^0^

Figure 2: Illustration of image rotation at different angles.


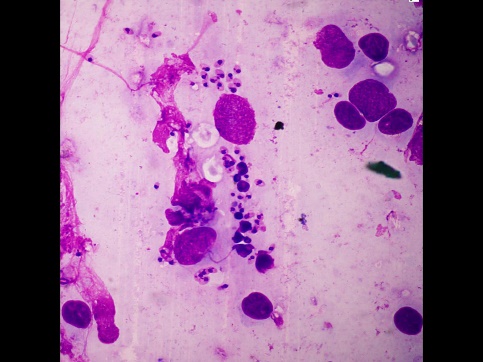

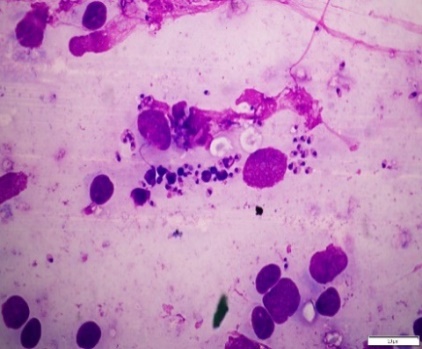


Original Image 90^0^rotated


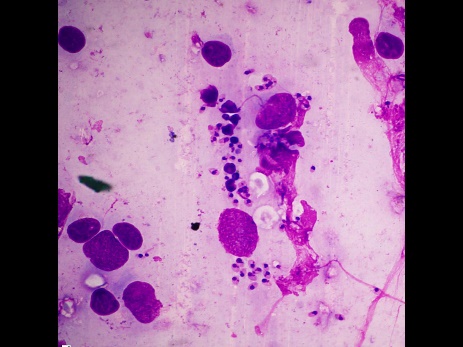

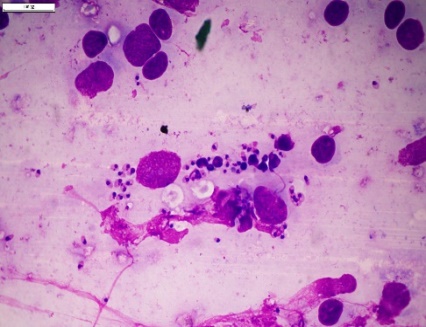


180^0^ rotated 270^0^ rotated

Figure 3: Images rotated with different angles.

1. **Object Detection Algorithms Used**

Object detection is a computer vision algorithm that is responsible for classifying and localization of objects in a given image and assigning each object instance to its corresponding class labels [1]. In this classification and localization task, the number of objects detected is not fixed; the number of output layers is variable. Hence, this problem can be solved by taking different regions of interest and classify the presence of the object in that region. The main drawback of this method is that one has to select a huge number of regions to localize an object of interest, which may have a different spatial location and aspect ratios within the image. Such methods are computationally intensive. Thus, several algorithms are developed to overcome these challenges in finding objects of interest. Yet the evaluation mechanisms for object detection tasks are still challenging. Currently used detection benchmarks to train and evaluate object detection models are discussed below.

**3.1 Detection Benchmarks of Object Detection**

Images for object detection were obtained from PASCAL VOC 2007-2012, MSCOCO datasets; this data set contains various images trained for object detection algorithms released in different years.

1. PASCAL Visual Object Class (VOC) dataset: The benchmarks, mainly containing the collection of data for object detection and annotation, are in XML format. It consists of 11,530 images with 27,450 regions of interest (ROI) annotated objects with 20 classes. It has PASCAL VOC 2007 and PASCAL VOC 2012 released formats.
2. MSCOCO dataset: Is another dataset container for object detection algorithms, it refers to common object contexts. It contains 333,000 images with 1.5 million object instances having 80 classes.

**3.2 State-of-the-Art Object Detection Models**

Recently, there have been various deep learning-based object detection models. State-of-the-art models to perform object detection tasks include RCNN [2], Fast RCNN [3], Faster RCNN [4], SDD [5], and YOLO [6]. These object detection models follow different procedures, commonly called single-stage detectors and region-based (two-stage) methods. The region-based method basically works by creating a region proposal from the input images and classifying the region proposal to a given class. The first region proposal (regions that have a high probability of containing the targeted object) method was RCNN, which is computationally expensive [7]. The latest one is faster RCNN, which follows the same steps as RCNN but uses a separate network to extract the feature vectors. This network type affects the accuracy and speed of detection.

There is no benchmark/standard comparison for the different object detection models to show which model is best, and yet it isn't easy to perform direct comparisons over the methods because it highly depends on the input image resolution, feature extractor type, speed, accuracy, and memory location they consume [8]. The best candidate model architectures for this study are discussed below.

1. **You Only Look Once (YOLO) V5 Architecture**

This model is one type of one-stage detector. The central concept of YOLO is to use the entire image as an input to the network and directly provide the position of bounding boxes with the category it belongs to. Basically, the model is composed of three main components. These are:

*Head:* predicts image features by generating bounding boxes and predicting categories. The confidence score implies accuracy prediction.

*Backbone:* is a CNN that aggregates and forms image features on different types of image granularity.

*Neck:* predicts image features to generate bounding boxes and predict categories. The classification accuracy under a specific condition is determined by the value of the confidence score.

Currently, YOLO is one of the one-stage detectors widely used in solving object detection problems. The model has five versions so far. It exhibits the highest Mean Average Precision (MAP) value in detecting small objects [43][9]. Figure 2.8 illustrates the YOLO V5 architecture. The basics of the YOLO V5 architecture are described below.


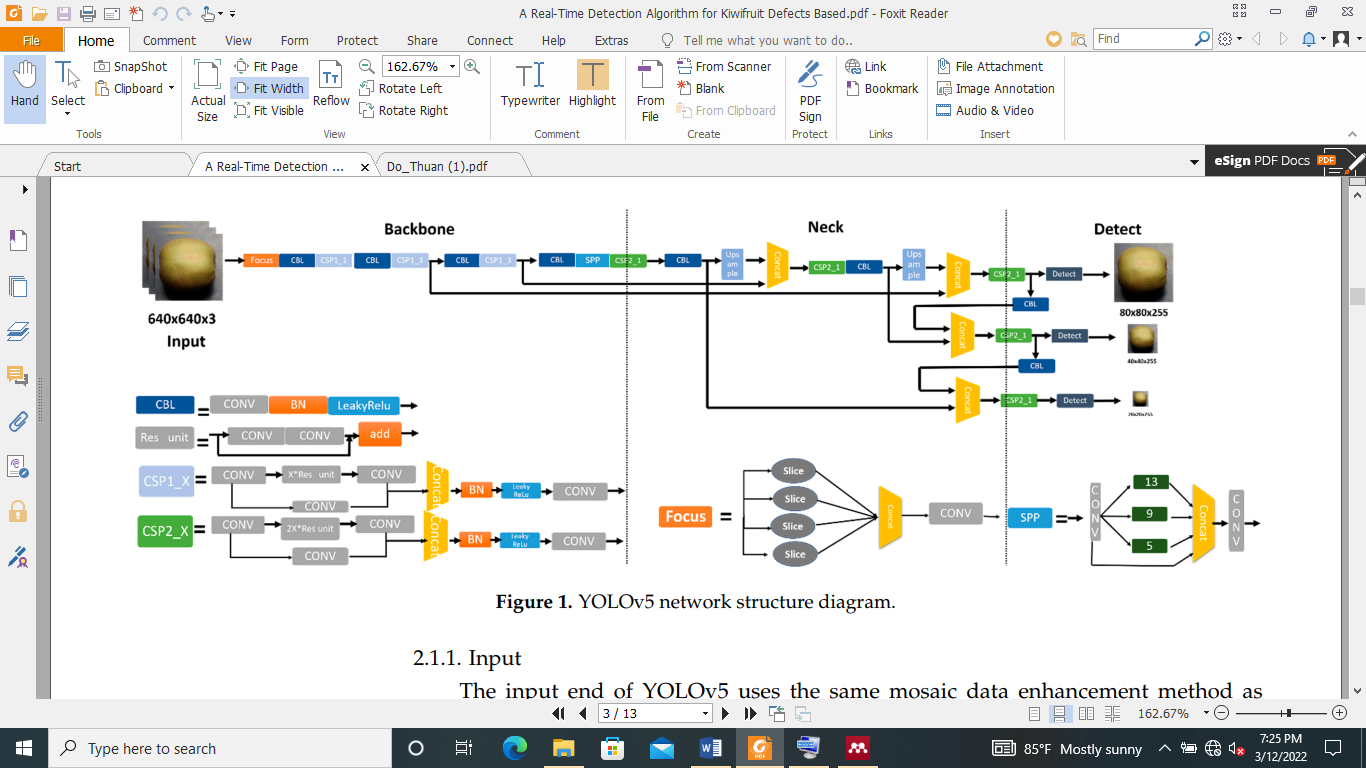


Figure 4: Overview of YOLO V5 architecture [10].

*Input:* provides image inputs and uses the same mosaic attachment method as YOLO V4. This enables it to perform better in small object detection. It also incorporates the function of adaptive anchor frame calculation. During each training, the value of the optimal anchor in different training sets is calculated adaptively.

*Backbone:* In this section, YOLO V5 consists of a focus structure to realize the slicing operation. For instance, if there is an original image input with 640x640x3 resolution, the slicer operation is used to obtain 320x320x12 feature maps. Then, after, using a convolution operation of 32 convolution kernels, it constructs the feature map of size 320x320x32.

*Neck:* is used to generate feature pyramids and enhance the model's detection ability at different scales. It has FPN-PAN structure. This FPN enhances the bottom-up path and improves the propagation of low-level features.

*Anchor Boxes*: In one-stage detection, anchor boxes can be adjusted based up on the size of the object to be detected. There are three types of boxes where an image could fit in it. These are square, rectangular and wide or rectangular and tall. Depending up on the aspect ratio and scale values, the anchor boxes can be adjusted according to the size of the target object automatically.

1. **Faster R-CNN Inception v2 Architecture**

The model is a two-stage detector using the inception v2 as a backbone. The first layer performs the convolutions and outputs fixed-sized feature maps. These feature maps are fed to the region proposal network to extract the regions. Then, the RPN and convolution layer output is fed into the ROI pooling layer. Finally, the ROI pooling layer outputs are fed to the classifier to classify further and localize the target object.


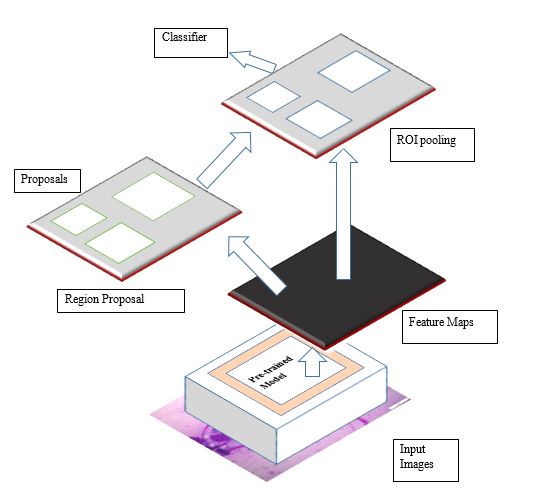


Figure 5 : Overview of Faster RCNN Architecture [11].

Figure 5 illustrates the RCNN architecture, and its basics are described below.

*Convolution layer:* Usually, the convolutional layer is the backbone of the model that generates the feature maps from the input image taken. In the current thesis work, the inception v2 2018 release is the backbone of the model.

*Region Proposal Network:* is responsible for generating the region proposals. It takes an image feature map of any size and provides a set of rectangular object proposals. It is a fully connected layer. A small sliding window is applied to convolutional feature maps to obtain the proposal. Each sliding window is mapped into a lower dimensional feature (256-d for ZF model or 512-d for VGG model). Then these lower dimensional features are fed to the two fully connected siblings, the classification layer and box-regression layer. These layers are sorted across all spatial locations where as the mini network operates in sliding window fashion on the whole image. At each sliding window, multiple proposals are predicted simultaneously. To elucidate, let K be the number of possible proposals generated at each location. Then, the classification layer outputs 2K scores that estimate the probability of Objectness or not for each proposal, and the regression layer outputs 4K encoding the coordinates of K boxes. These K proposals are known as anchors. Anchors are centered at the sliding window in question, and it is related to the aspect ratio and a scale; the default value is 3x3, yielding K = 9 anchors at each sliding position [11].

In Faster RCNN, there are translation invariant anchors, both in terms of anchors and functions that compute proposal relative to the anchor. This property reduces the model size because if an object translates, the same function has to predict the location of the proposal in either location. Hence, the translation-invariant property is guaranteed. The other important point is about the multi-scale anchors as a regression reference. There are two methods to do this; the first one is based on feature pyramids/images. The second way is using a sliding window of multi-scale on a feature map. The anchor-based method of RPN is built on a pyramid of anchors, which is a cost-effective way because it classifies and regress the boxes with respect to the anchors boxes with different scales and aspect ratio. Hence, a simple convolutional feature can be computed because of the multi-scale design based on anchors. It implies that such a multi-scale anchor design is a key component for sharing features without extra cost for addressing scales and is cost-efficient. The region proposal network of Faster RCNN is illustrated in Figure 6.


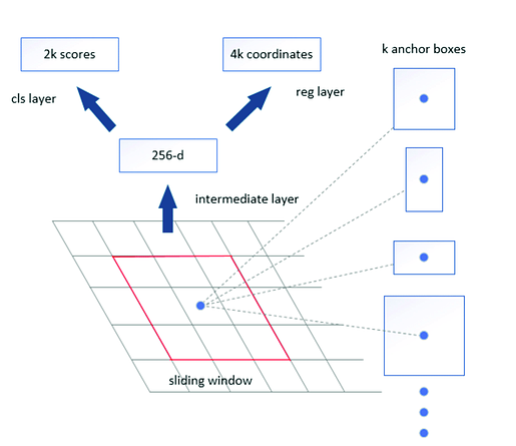


Figure 6: Region Proposal Network of Faster RCNN [11].

*ROI Pooling Layer:* this is a neural network layer used to speed up both training and testing phases with maintaining high accuracy. First proposed by Ross Grishik in 2015 [46][12], it takes two inputs: one is a fixed-size feature map, and the second one is from the region proposal network. The fixed-size feature maps are obtained from the deep convolutional neural network with several convolutions and maximum pooling boxes. The output from the RPN is an Nx5 matrix representing a list of regions of interest. The first column represents the image index and the remaining four columns represent the top, left, right, and bottom corners of the region proposals. For every ROI from the input list, the ROI pooling layer takes a section if the input feature map corresponds to it and scales it to some predefined size. Two steps do the scaling. First, the region proposals are divided into sections of equal size (i.e., the number of sections is the same as the dimension of the output). Secondly, the largest value in each section is obtained. Finally, these maximum values are copied to the output buffer. Hence, it forms a list of rectangles with different sizes in which the corresponding feature maps with fixed size can be obtained. As a result, this speed up the train-test processing speed, it makes end-to-end training possible, and it allows re-using the feature maps from the CNN.

*Classifier:* During the utilization of object detection-based deep learning algorithms, various classifiers are deployed to perform classification tasks during training phases. ADAM and Softmax classifiers are the most commonly referred classifiers.

*Anchor Generation Methods:* In two-stage detection, anchor boxes can be adjusted based on the size of the object detected. There are three types of boxes where an image could fit in it. These are square, rectangular, and wide, or rectangular and tall. Depending up on the aspect ratio and scale values, the anchor boxes can be adjusted according to the size of the target object.

*The backbone of Faster RCNN Model*: Faster RCNN model with different feature extractors was developed. Depending on the application it is needed for, the model is used to train object detection models. Whenever there is a situation to decide whether to perform convolutions with a fixed kernel size in each block of a pooling layer, most of the neural networks developed perform convolution with fixed-size kernels. Instead of choosing which filter size to perform the convolution, performing all convolutions and concatenating the results could reduce the computational costs. A neural network performing this task is called the inception module. These inception modules are connected and form the inception network. Four versions have recently been released, and they are modified and integrated with other networks. In the current thesis work, the inception v2 architecture is used as a feature extractor. It is known that choosing the appropriate feature extractor could actually depend on several factors like dataset type, memory consumption, time consumption, etc. It is commonly used and accepted by many researchers in its performance. According to a previous study, the inception v2 model was able to achieve a better accuracy tradeoff among the other versions of small object detection feature extractors [8]. Inception v2 feature extractor can actually learn the essential features of the target objects and successfully extract the necessary features.

1. **Single Shot Detector Model Architecture**

The single-shot detector (SSD) is one of the one-stage detectors. It consists of two components: the backbone and the head. The backbone is composed of pre-trained networks used as feature extractors. The head consists of one or more convolutional layers added to the backbone to predict the classes and location of the object. SSD divides the image into grid cells, and each grid cell is responsible for detecting objects. Not all objects have the same shape, and multiple objects may be present in one grid cell. SSD handles these by defining anchor boxes and adjusting zoom levels and aspect ratios. During training, the anchor boxes are matched with bounding boxes for each ground truth object in an image; the highest degree of overlap with the target object indicates the location and class of the object. Aspect ratios determine the shape of the anchor boxes, and the zoom level values adjust the scale of the anchor boxes relative to the grid cell (see also Figure 7 below).


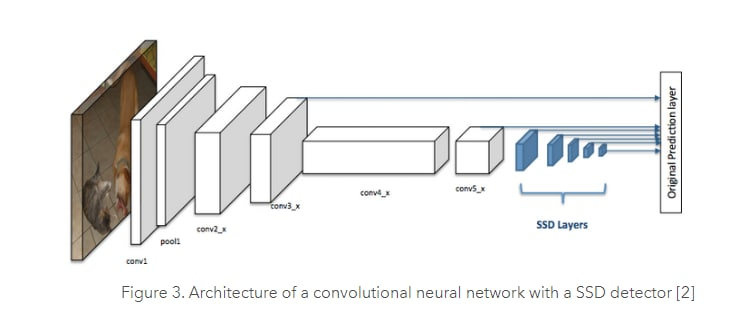


Figure 7: Architecture of a single shot detector [13].

**Hyper-parameters and Optimization**

In machine learning, there are two types of parameters. These are machine-learnable parameters and Hyper-parameters. The machine learnable parameters are the ones that the algorithm learns on its own from the dataset given, whereas the hyper-parameters are the ones that the data scientist or the engineers assign to a specific value for the sake of controlling the way the algorithm performs and to tune the performance of the model. The hyper-parameters optimized in the current thesis work are described as follows.

*Learning Rate*: is a hyper-parameter used to control the rate at which the parameter is estimated. It is also known as step size: it tries to multiply the gradient with the scalar to determine the next point [14].

*Batch size:* is a hyper-parameter used to determine the number of samples to work through before updating the internal model parameter, and it depends on the size of the dataset. Training sets can be divided into one or more batches. When all the training samples are used to create the batch size, it is called *Batch Gradient Decent.* But, if the batch size is equivalent to the size of one sample, it is known as *Stochastic Gradient Decent*. Stochastic Gradient Decent is an optimization tool; it tries to find the set of internal model parameters that perform well against some performance measure. When the size of the sample is in between the size of the dataset and one sample, it is known as *Mini-Batches Gradient Decent*. The most popular mini-batches are in between 10 and 1000 [48][14].

*Step/Iterations and Epochs*: is the number of batches between the graphs in which at each step where the weights are updated in a direction that minimizes the loss. The number of steps is specified based up on the number of images, the batch size, and the value of epoch (see Equation 2.1). As the batch size increases, the number of steps should decrease. The same is true for the total training dataset and epoch. In fact, epoch is randomly chosen and selected after performing small experiment on whether the model learns the important features based upon the specified time limits. Likewise, the batch size is also determined based up on the result of the small experiment.

$Step=\frac{\left( no. of images*epoch \right)}{Batch size}$ (2.1)

**References**

[1] X. Wu, D. Sahoo, and S. C. H. Hoi, 'Recent advances in deep learning for object detection', *Neurocomputing*, vol. 396, pp. 39–64, 2020, doi: https://doi.org/10.1016/j.neucom.2020.01.085.

[2] R. Girshick, J. Donahue, T. Darrell, and J. Malik, 'Rich feature hierarchies for accurate object detection and semantic segmentation', *Proc. IEEE Comput. Soc. Conf. Comput. Vis. Pattern Recognit.*, pp. 580–587, 2014, doi: 10.1109/CVPR.2014.81.

[3] L. Jiao *et al.*, 'A survey of deep learning-based object detection', *IEEE Access*, vol. 7, no. 3, pp. 128837–128868, 2019, doi: 10.1109/ACCESS.2019.2939201.

[4] J. Hung and A. Carpenter, 'Applying Faster R-CNN for Object Detection on Malaria Images', *IEEE Comput. Soc. Conf. Comput. Vis. Pattern Recognit. Work.*, vol. 2017-Janua, 2017, doi: 10.1109/CVPRW.2017.112.

[5] S. Jia *et al.*, 'Object Detection Based on the Improved Single Shot MultiBox Detector', *J. Phys. Conf. Ser.*, vol. 1187, p. 42041, Apr. 2019, doi: 10.1088/1742-6596/1187/4/042041.

[6] S. Chibuta and A. C. Acar, 'Real-time Malaria Parasite Screening in Thick Blood Smears for Low-Resource Setting.', *J. Digit. Imaging*, vol. 33, no. 3, pp. 763–775, Jun. 2020, doi: 10.1007/s10278-019-00284-2.

[7] R. Girshick, J. Donahue, T. Darrell, and J. Malik, 'Rich Feature Hierarchies for Accurate Object Detection and Semantic Segmentation', in *2014 IEEE Conference on Computer Vision and Pattern Recognition*, 2014, pp. 580–587, doi: 10.1109/CVPR.2014.81.

[8] J. Huang *et al.*, 'Speed/accuracy trade-offs for modern convolutional object detectors', Nov. 2016.

[9] D. Thuan, 'Do Thuan EVOLUTION OF YOLO ALGORITHM AND YOLOV5: THE STATE-OF-THE-ART OBJECT DETECTION ALGORITHM EVOLUTION OF YOLO ALGORITHM AND YOLOV5: THE STATE-OF-THE-ART OBJECT DETECTION ALGORITHM', 2021, [Online]. Available: https://api.semanticscholar.org/CorpusID:235341555.

[10] J. Yao, J. Qi, J. Zhang, H. Shao, J. Yang, and X. Li, 'A Real-Time Detection Algorithm for Kiwifruit Defects Based on YOLOv5', *Electronics*, vol. 10, no. 14. 2021, doi: 10.3390/electronics10141711.

[11] S. Ren, K. He, R. Girshick, and J. Sun, 'Faster R-CNN: Towards Real-Time Object Detection with Region Proposal Networks', *IEEE Trans. Pattern Anal. Mach. Intell.*, vol. 39, no. 6, pp. 1137–1149, 2017, doi: 10.1109/TPAMI.2016.2577031.

[12] R. Girshick, 'Fast r-cnn', Apr. 2015, doi: 10.1109/ICCV.2015.169.

[13] W. Liu *et al.*, 'SSD: Single Shot MultiBox Detector BT - Computer Vision – ECCV 2016', 2016, pp. 21–37.

[14] L. Smith, 'A disciplined approach to neural network hyper-parameters: Part 1 -- learning rate, batch size, momentum, and weight decay', Mar. 2018.
